# Slow EEG potentials as predictors of cognitive impairment in patients with clinical high risk for schizophrenia

**DOI:** 10.1192/j.eurpsy.2025.2115

**Published:** 2025-08-26

**Authors:** M. V. Slavutskaya, A. V. Pavlov, A. P. Dzhem, A. Y. Komarova, M. A. Omelchenko, I. S. Lebedeva

**Affiliations:** 1 Department of the highest nervouse activity, Lomonosov Moscow State University; 2 Laboratory of neuroimaging and multimodal analysis; 3 Department of adolescent psychiatry, Mental Health Research Center, Moscow, Russian Federation

## Abstract

**Introduction:**

Cognitive deficits in schizophrenia are associated with impaired predictive processes, however, the neural mechanisms of these impairments at the early stages of the disease are poorly understood. A modified memory-guided saccade task can be informative for studies in this field. The contingent negative variation (CNV) slow negative potentials (SNP1, 2, 3 waves) in 1000-ms interval before a memory-guided response are considered to be neural correlates of attention, memory, motor, and inhibitory predictive processes.

**Objectives:**

We aimed to assess the CNV-type slow negative event-related potentials (ERP) during the latent period before the signal to perform remembered saccades in patients with clinical high risk (CHR) for schizophrenia.

**Methods:**

An electroencephalogram (EEG) from 24 electrodes and electrooculogram of horizontal eye movements were recorded in 16 patients with CHR and 18 healthy controls. The participants had to remember the location of a peripheral stimulus (PS, 150ms) and perform a saccade or antisaccade (50% probability) when the central fixation stimulus (CFS) was turned off after a delay period of 2800–3000 ms. The СFS shape (cross or circle) defined a motor response type: saccade or antisaccade.

**Results:**

The task performance (assessed based on response latency and errors) was worse in CHR patients compared to controls. In the antisaccade condition, SNP1 was faster in CHR patients compared to controls possibly reflecting attention deficits in CHR patients. The SNP1 amplitude peaks were equally distributed across the EEG leads in CHR patients but were located predominantly in frontal and central leads in controls. Diffuse representation of the amplitude peaks may reflect a compensatory involvement of posterior temporal and parietal-occipital cognitive control networks at the early stages of schizophrenia. At the last 300 ms of the delay period, the late SNP3 wave was shorter before memory-guided antisaccades compared to saccades only in patients. This may reflect the violation of predictive attention processes as well as proactive inhibition deficits, that are well-known in schizophrenia, in CHR patients.

**Conclusions:**

Based on our data we consider the SNP1 and SNP3 components in the memory-guided saccade task to be potentially significant neurobiological markers of cognitive control at the early stages of schizophrenia.

The research was carried out within the state assignment of Ministry of Science and Higher Education of the Russian Federation (theme No.121032500081-5 and No.AAAA-A19-11904049098-9).

**Disclosure of Interest:**

None Declared

